# Identification of skewed X chromosome inactivation using exome and transcriptome sequencing in patients with suspected rare genetic disease

**DOI:** 10.1186/s12864-024-10240-2

**Published:** 2024-04-16

**Authors:** Numrah Fadra, Laura E Schultz-Rogers, Pritha Chanana, Margot A Cousin, Erica L Macke, Alejandro Ferrer, Filippo Pinto e Vairo, Rory J Olson, Gavin R Oliver, Lindsay A Mulvihill, Garrett Jenkinson, Eric W Klee

**Affiliations:** 1 Quantitative Health Sciences, Mayo Clinic, Rochester, MN USA; 2 Center for Individualized Medicine, Mayo Clinic, Rochester, MN USA; 3 Division of Hematology, Mayo Clinic, Rochester, MN USA; 4 Department of Clinical Genomics, Mayo Clinic, Rochester, MN USA

**Keywords:** Skewed X chromosome inactivation, Non-random skew, Exome sequencing, Transcriptome, Rare genetic disease, Escape, Expression

## Abstract

**Background:**

X-chromosome inactivation (XCI) is an epigenetic process that occurs during early development in mammalian females by randomly silencing one of two copies of the X chromosome in each cell. The preferential inactivation of either the maternal or paternal copy of the X chromosome in a majority of cells results in a skewed or non-random pattern of X inactivation and is observed in over 25% of adult females. Identifying skewed X inactivation is of clinical significance in patients with suspected rare genetic diseases due to the possibility of biased expression of disease-causing genes present on the active X chromosome. The current clinical test for the detection of skewed XCI relies on the methylation status of the methylation-sensitive restriction enzyme (Hpall) binding site present in proximity of short tandem polymorphic repeats on the androgen receptor (AR) gene. This approach using one locus results in uninformative or inconclusive data for 10–20% of tests. Further, recent studies have shown inconsistency between methylation of the AR locus and the state of inactivation of the X chromosome. Herein, we develop a method for estimating X inactivation status, using exome and transcriptome sequencing data derived from blood in 227 female samples. We built a reference model for evaluation of XCI in 135 females from the GTEx consortium. We tested and validated the model on 11 female individuals with different types of undiagnosed rare genetic disorders who were clinically tested for X-skew using the AR gene assay and compared results to our outlier-based analysis technique.

**Results:**

In comparison to the *AR* clinical test for identification of X inactivation, our method was concordant with the *AR* method in 9 samples, discordant in 1, and provided a measure of X inactivation in 1 sample with uninformative clinical results. We applied this method on an additional 81 females presenting to the clinic with phenotypes consistent with different hereditary disorders without a known genetic diagnosis.

**Conclusions:**

This study presents the use of transcriptome and exome sequencing data to provide an accurate and complete estimation of X-inactivation and skew status in a cohort of female patients with different types of suspected rare genetic disease.

**Supplementary Information:**

The online version contains supplementary material available at 10.1186/s12864-024-10240-2.

## Background

In females, X chromosome inactivation (XCI) provides dosage compensation for genes on the X chromosome by random inactivation of one of two copies of the X chromosome. The process ensures that the expression of genes on the X chromosome occurs at levels comparable to that of chromosomally XY males [1]. During early embryogenesis, the choice of which of the two alleles is inactivated is generally independent from the effects of parental origin. In this case, there will be equal probability of either parental X chromosome being silenced, giving rise to an even proportion of cells expressing the inactive X from either parent [2]. After XCI has been established, the inactive X is subsequently inherited by all daughter cells during mitosis. However, not all females have an even ratio of cells expressing the active copy of X from either parent and a number of different mechanisms can result in such skewed ratios [3]. The so-called non-random or skewed XCI (X-skew) can arise by chance or due to primary and secondary genetic factors. Primary X-skew involves the presence of variants on genes involved in the process of XCI, for example, *XIST* (X inactive specific transcript) which prevent the cell from inactivating the X chromosome harboring those variants. XCI is initiated in humans by the expression of *XIST*, a gene encoding a long non-coding RNA, initiating a cascade of epigenetic modifications that spreads *in cis* on the X chromosome to be inactivated resulting in the formation of a dense heterochromatin called a Barr body [4]. Skewing resulting from secondary genetic factors often occurs in females harboring deleterious variants, unfavored polymorphisms, tissue-specific gene imprinting, and large structural abnormalities on the X chromosome [2, 4]. For example, in females with Duchenne Muscular Dystrophy (DMD), a severe X-linked disorder, reportedly all show skewed XCI resulting from the inactivation of the normal parental copy and preferential expression of the X chromosome harboring pathogenic variants in the *DMD* gene [1, 5]. Often, when a female carrier of an X-linked disorder does not show the suspected phenotype, a skewed XCI pattern reveals preferential inactivation of the diseased allele and expression of the wild type. The reverse is possible if the X linked carrier displays a phenotype which can be explained by skewed activation of the mutated allele. Skewed patterns of XCI are common in humans, with estimated prevalence around 25% in adult females [1, 6].

The X chromosome consists of over 800 protein-coding genes and roughly two-thirds of them have reported pathogenic variants associated with X-linked diseases. There are over 141 known X-linked genes associated with intellectual disability [4]. The majority of patients who are carriers of deleterious variants for X-linked intellectual disorders present with notably skewed XCI patterns [7]. The X chromosome is also known to be enriched in hormone-related genes associated with hormonal carcinogenesis [8]. Tumor suppressor genes harboring deleterious variants on the X chromosome may drive tumor progression by disrupting gene expression of genes relevant to normal growth and development [9]. Studies conducted on prenatal samples for the detection of XCI patterns in multiple pedigrees reported the presence of skewed XCI in female carriers of heterozygous X-linked deletions affirming the prognostic value for analyzing patterns of XCI [10]. These studies affirm the importance of XCI in rare disease diagnostics as well as the prognostic and diagnostic value of XCI in clinical practice.

Currently, the only clinically validated test for evaluation of XCI patterns relies on the methylation status of the methylation-sensitive restriction enzyme (Hpall) binding site present within 100 base pairs of the short tandem repeats (STR) on the first exon of the human androgen receptor (*AR*) gene. When the polymorphic trinucleotide CAG repeat differs in length on the parental alleles, gel electrophoresis of the polymerase chain reaction (PCR) amplified product for this region of the gene identifies distinct bands for each parental product. However, because the assay relies on the presence of different polymorphic repeat size, it cannot report a result in 10–20% of cases with equal length “CAG” repeats on both parental alleles. Further, the assay assumes that the methylation status of a single locus reflects the chromosome-wide inactivation status of the X chromosome. A comparative analysis of the *AR* gene methylation assay and direct measurement of allele-specific expression of distinct heterozygous loci using quantitative reverse transcription-polymerase chain reaction (RT-PCR) revealed discordant results indicating that the methylation of *AR* locus alone does not always accurately reflect the expression along the entirety of the X chromosome [6].

Previous studies have made use of NGS data in the X-skew paradigm and reported the presence of X-skew as a common observation in the general population [1], however the accuracy of the study required sequenced information from parental samples which may not always be readily available or maybe unaffordable due to increased costs of trio/familial sequencing and analysis in case of patient samples presenting to the clinic for genetic testing. Other studies made use of outlier analysis techniques by generating a transcriptome profile for healthy controls and patients with muscle disorders for investigating transcriptome wide aberrant events [11]. More recently, Lappalainen et al [12] developed an outlier analysis technique using healthy controls in the context of studying regulatory variation at the population level by applying statistical methods for measuring expression outliers at allelic levels. However, these methods are limited in their implementation for determining skewed levels of XCI in female individuals with suspected phenotypes for rare diseases as they lack (a) a reference model which can be universally used to calculate an outlier skew at the variant level for positions not represented within the population (b) an integrated approach combining the application of outlier analysis techniques using whole exome and transcriptome sequencing without the need for pedigree information. To address these concerns, we developed an approach that integrates concepts of outlier detection and X-skew measurement using genotype information for heterozygous loci from exome sequencing (ES) as a guide for mining the expression of corresponding loci within the transcriptome thereby providing a chromosome-wide measure of skewness along the X chromosome. Herein, we present a novel and effective outlier-based method for the identification of X-skew using whole blood in 81 female patients with different clinical implications for genetic testing by modeling X-skew in a healthy cohort of 135 females from The Genotype-Tissue Expression (GTEx) consortium [13]. We leveraged exome and transcriptome data for determining XCI status and assess our results in comparison to existing clinical-grade testing for X-skew in 11 female patients. Finally, we discuss the implications for identifying skewed XCI in clinical practice for patients with rare genetic disorders using variant information from multiple omic sources (DNA and RNA) including two case studies using patient data.

## Results

### Reference, validation and evaluation cohorts

The study established a method for detection of X-skew status using a reference cohort of 135 females from the GTEx consortium [13]. We validated the method using an outlier-based analysis technique on 11 patients who were evaluated for clinical X-skew testing using the *AR* gene assay. Finally, we applied the method on 81 additional female patients. In total, the study made use of 92 female patients presenting to the clinic with phenotypes consistent with different types of rare genetic disorders with no known genetic diagnosis.

### Evaluation of XCI status in reference cohort (GTEx)

**Fig. 1:**
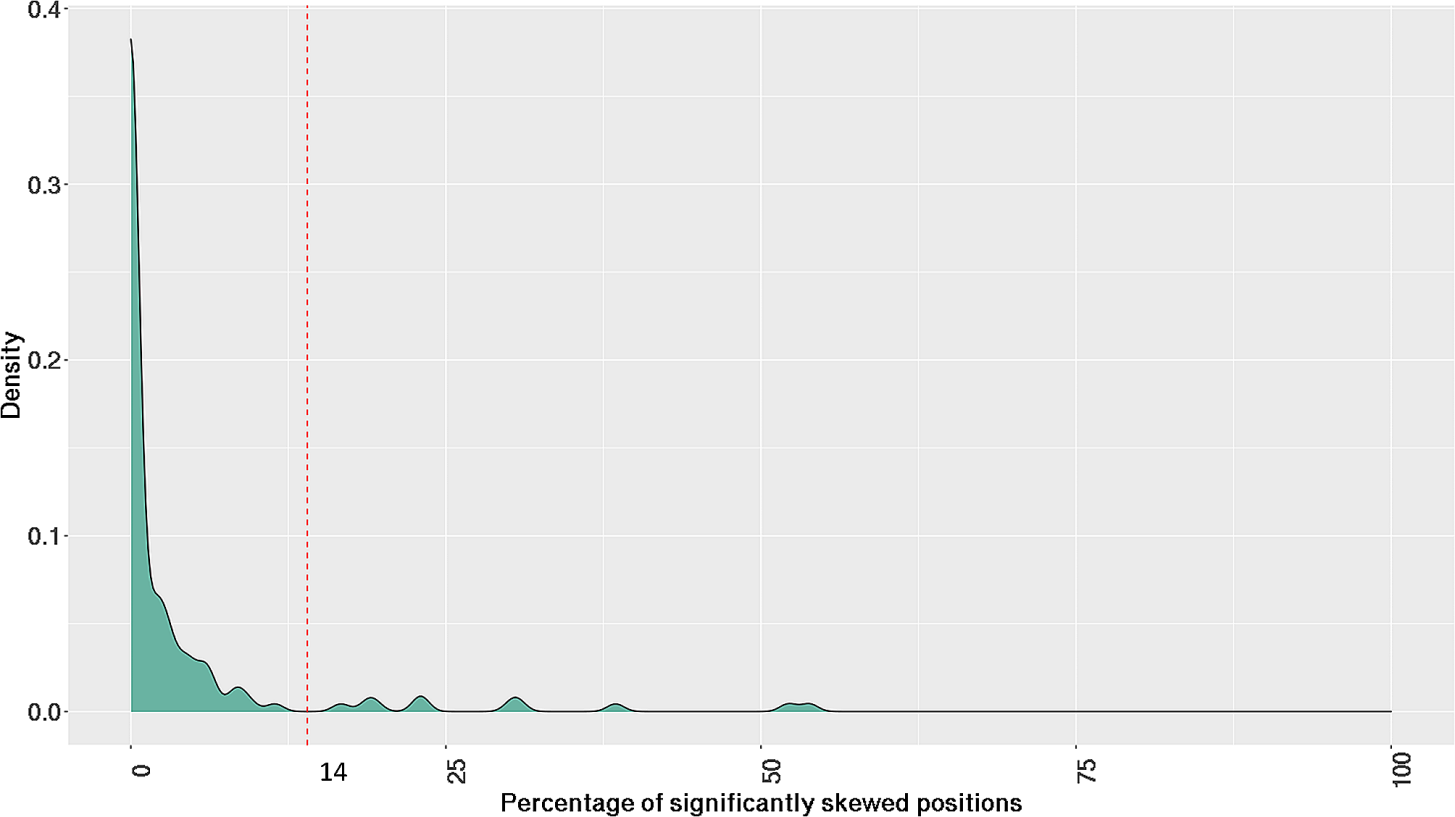
Density plot for 135 GTEx reference females displaying the percentage of statistically skewed variant positions when tested on a per sample basis. The red dotted line indicates the selected threshold for defining individuals with X-skew versus patients without X-skew. The threshold was selected to fall within the range of the reported frequency of X-skew in the general population and aligned to a local minimum in the density plot

135 females from the GTEx consortium were used for building a reference model that represents the distribution of allele counts on the X chromosome in the general female population. To determine an appropriate threshold to assign a status of skewed or random XCI for a sample, we evaluated these 135 GTEx female samples on a case-by-case basis against the reference model built using the same set of females. Figure 1 shows the density plot for 135 female samples from the GTEx consortium with a right skewed distribution. The right skewed density peaks reflect a subset of samples with a higher proportion of variants showing significantly biased expression. The right skewed samples can be segmented using the drop in density at 14% establishing a plausible threshold aligned to a local minimum on the plot whereby a sample is called skewed if > 14% of variants have significantly biased allelic expression (Fig. 1). 124/135 females in the GTEx population do not meet our threshold for X-skew indicating that most of these samples (> 90%) express both parental alleles and show random patterns of X inactivation. A 14% threshold in the reference population results in ∼ 8% of females (11/135) identified with skewed XCI patterns. Although the applied threshold is governed by the reference population used within this study, the results for skewed XCI observed in the general population are consistent with previously published studies [1, 6].

### Validation of computed XCI status using clinical XCI testing in 11 female patients suspected of rare genetic disorders

**Fig. 2:**
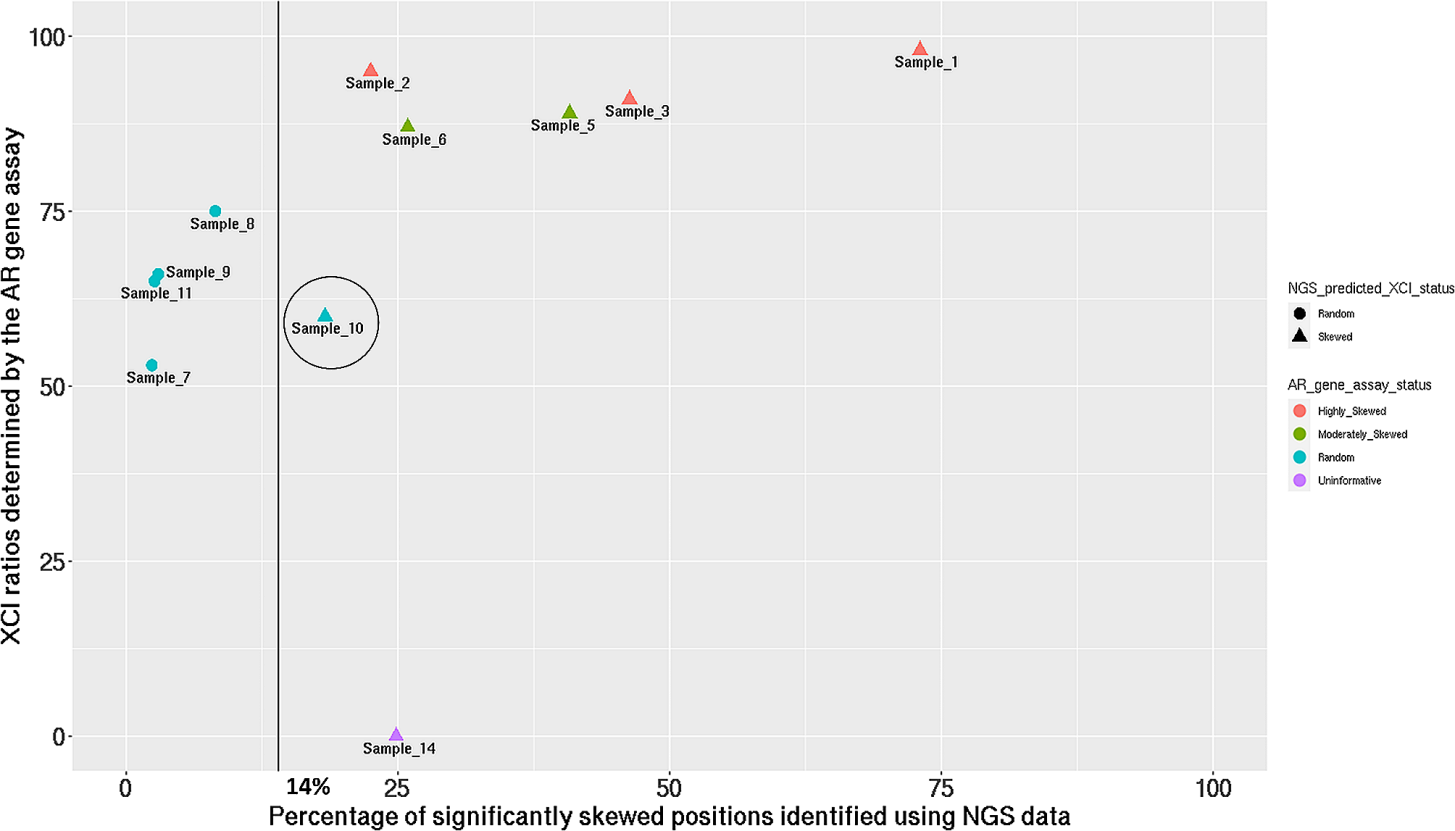
Dot plot representing XCI ratios calculated by the *AR* gene assay on the Y axis and the percentage of significantly skewed positions calculated using NGS data on the X axis for the 11 female patients. The sample encircled represents the patient in which the *AR* gene assay reported random patterns of XCI, and our analysis identified skewed XCI

We compared the predicted level of XCI skew with clinical XCI testing performed at Greenwood Genetic Center for 11 female patients. Clinical testing reported 5 patients with skewed XCI patterns (3 high, 2 moderate), 5 patients with random XCI, and an inconclusive result for 1 patient. 9 results were concordant with our predicted XCI skew status, including all 5 skewed patients and 4 random XCI patients, as shown in Table 1; Fig. 2. One discordant result was predicted as skewed XCI by our method, whereas the clinical assay reported random XCI (Table 1; Fig. 2). Finally, clinical testing reported an inconclusive result for 1 patient (Table 1), due to the presence of undistinguishable length of polymorphic “CAG” repeats within exon 1 of *AR* on each parental allele. For this patient, our method identified a skewed pattern of XCI based on NGS data showing biased allelic expression of over 25% of expressed variants; a nearly 2-fold increase from our empirically derived threshold for X-skew (Table 1; Fig. 1). Table 2 shows a confusion matrix for the comparison between our method and the clinical test for XCI. The concordance rate or percentage positive agreement between the clinical test and the method outlined in this study is 90% (95% CI = 0.55,0.99).

**Table 1:** XCI status results for 11 females assessed for XCI skew clinical testing using the *AR* gene assay and our internally developed method using NGS

Sample	XCI status from the AR gene assay (X inactivation ratios)	Percentage of variants showing skew with *P*-value < 0.05	XCI Status Identified by NGS
Sample_1*	Highly skewed (98:2)	73.04	Skewed
Sample_2*	Highly skewed (95:5)	22.43	Skewed
Sample_3*	Highly skewed (91:9)	46.32	Skewed
Sample_5*	Moderately skewed (89:11)	40.85	Skewed
Sample_6*	Moderately skewed (87:13)	25.93	Skewed
Sample_7*	Random (53:47)	2.35	Random
Sample_8*	Random (75:25)	8.24	Random
Sample_9*	Random (66:34)	2.94	Random
Sample_11*	Random (65:35)	2.63	Random
Sample_10**	Random (60:40)	18.37	Skewed
Sample_14***	Uninformative (NA)	25.84	Skewed

* Results using NGS data that show agreement with the clinical assay, ** Results using NGS data that differ from the clinical assay, *** Result using NGS data for the individual uninformative by the AR gene assay

**Table 2:** Confusion matrix for comparison of X skew testing using the clinical *AR* gene assay and the internally developed NGS method outlined in the proposed study

Method		AR gene assay		
		Random	Skewed	Uninformative
NGS	Random	4	0	0
	Skewed	1	5	1

**Fig. 3:**
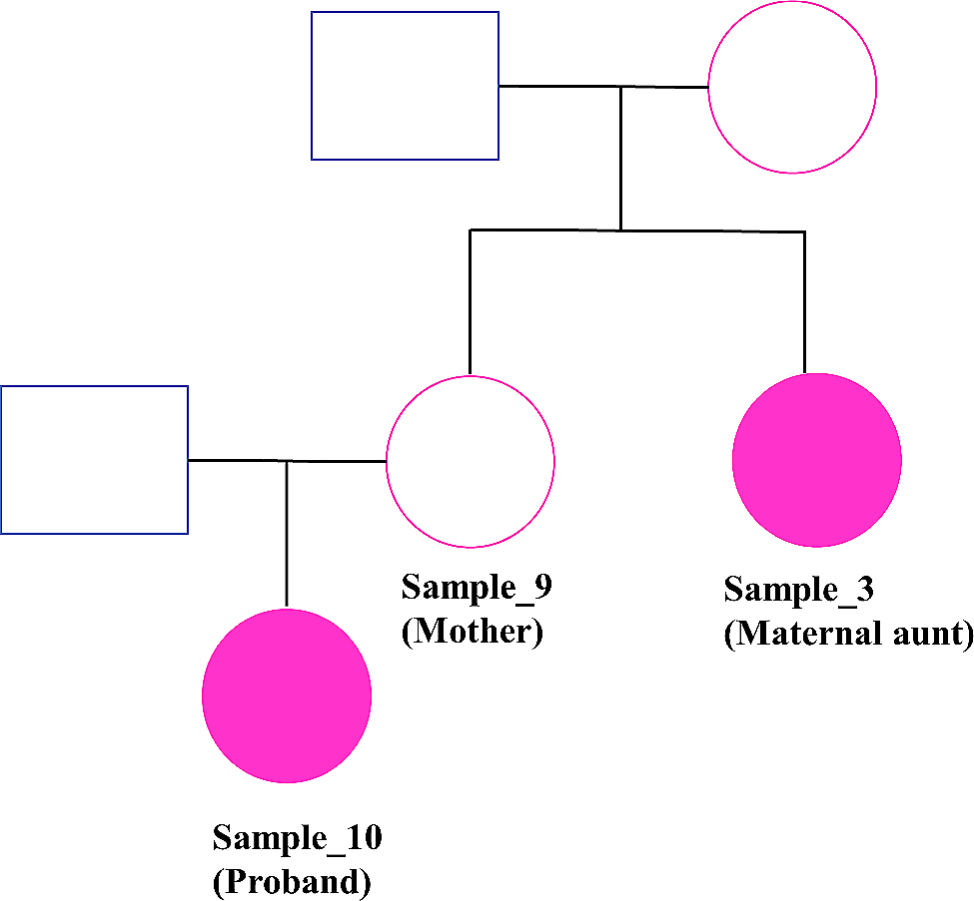
Progeny graph for Sample_10 (proband) for presence of Dubowitz syndrome phenotype in maternal aunt and proband, both of which were identified to show skewed patterns of XCI from NGS data

The XCI results from our method are discrepant with the clinical test for Sample_10 (Table 1; Fig. 2). We observed an increased proportion of variants in this sample along the X chromosome that show skewed expression relative to the general population. Sample_10 has family members who were also tested clinically (Tables 1 and 3; Figs. 2 and 3). Sample_10’s maternal aunt (Sample_3) tested positive for skewed XCI, while her mother (Sample_9) tested negative for skewed XCI as per the clinical assay (Tables 1 and 3; Figs. 2 and 3). Interestingly, this family presents with Dubowitz Syndrome phenotype in the proband (Sample_10) and maternal aunt (Sample_3), but this phenotype is absent in the mother (Sample_9) (Fig. 3 ).

**Table 3:** Summary of results for related female patients assessed for X-skew using the internally developed NGS method in the proposed study and the clinical *AR* gene assay

Sample Name (Type)	X-skew results from the AR gene assay	X-skew results from NGS
Sample_10 (Proband_1)	Random	Skewed
Sample_9 (Mother_1)	Random	Random
Sample_3 (Maternal_aunt_1)	Skewed	Skewed

### Evaluation of computed XCI status in 81 additional female patients suspected of rare genetic disorders

We applied the proposed method for evaluating XCI in 81 additional female patients clinically tested for suspected rare genetic diseases and enrolled in a research study. The percentage of significantly skewed variant positions along the X chromosome within coding regions excluding the PAR regions was computed for all samples (Supplementary Table 1) [1, 6]. Supplementary Fig. 3 displays the density plot for the 81 female patients in our rare disease cohort. Similar to the frequency of skew observed in the general population, applying our threshold (Supplementary Fig. 3) identified ∼ 10% (8/81) patients with skewed XCI (Table 4). Table 4 displays the percentages of variants showing biased expression in these 8 females identified as skewed XCI using our method. Supplementary Table 1 provides information on the XCI status, percentage of significantly skewed variants observed in the validation (*N* = 11) and the aforementioned application rare disease cohort (*N* = 81).

**Table 4:** The percentages of variants showing biased expression in 8 females inferred to present with skewed XCI from the application cohort (*N* = 81)

Sample_ID	Percentage of variants showing skew with *P*-value < 0.05	XCI Status Identified using NGS
Sample_15	22.06	Skewed
Sample_17	17.24	Skewed
Sample_18	16.67	Skewed
Sample_19	15.38	Skewed
Sample_20	14.93	Skewed
Sample_21	14.49	Skewed
Sample_22	14.46	Skewed
Sample_23	13.56	Skewed

### Comparison of position, gene and global models for significantly skewed positions in validation and application cohorts

**Fig. 4:**
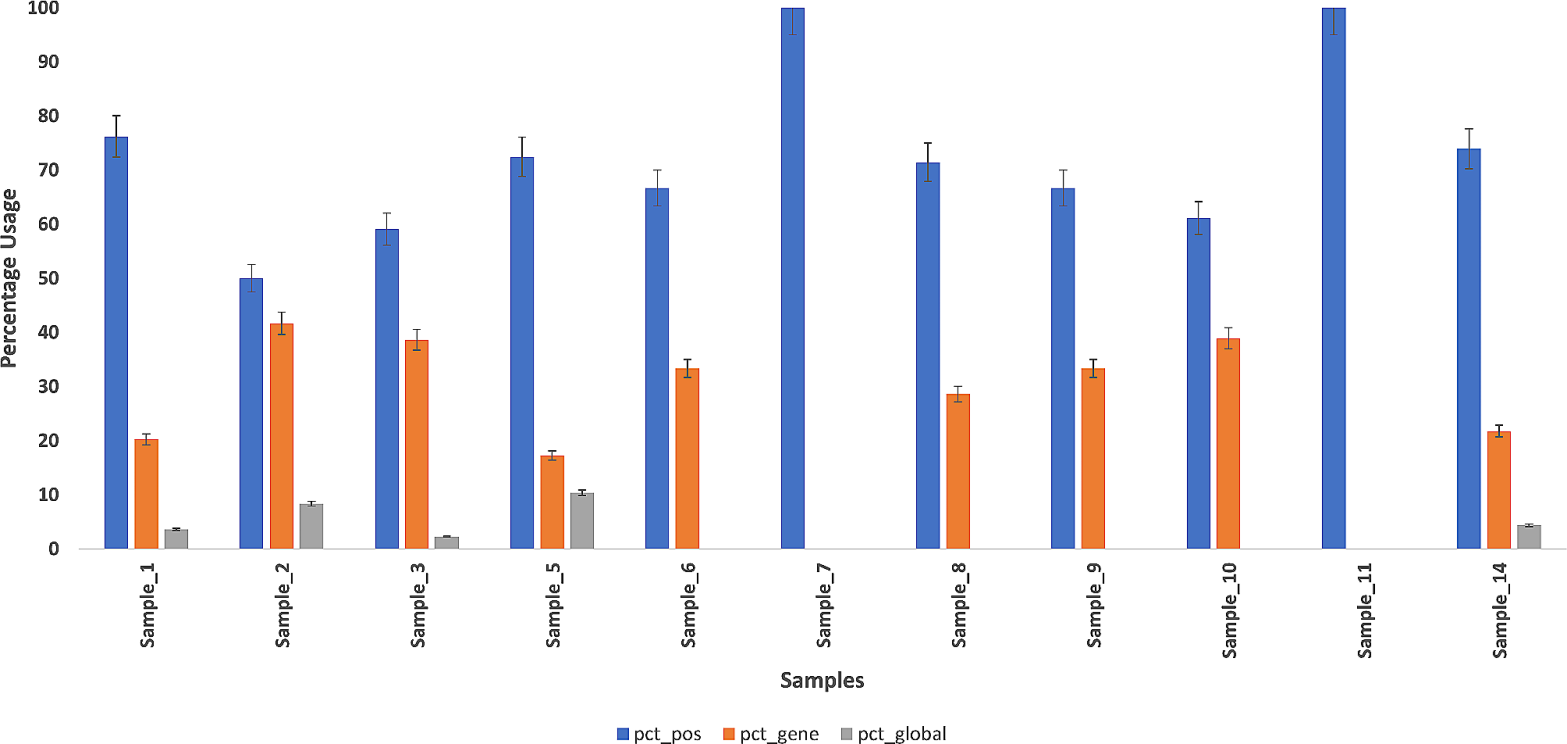
Percentage of usage across the position (blue), gene (orange), global (grey) models for significantly skewed positions in 11 individuals

**Fig. 5:**
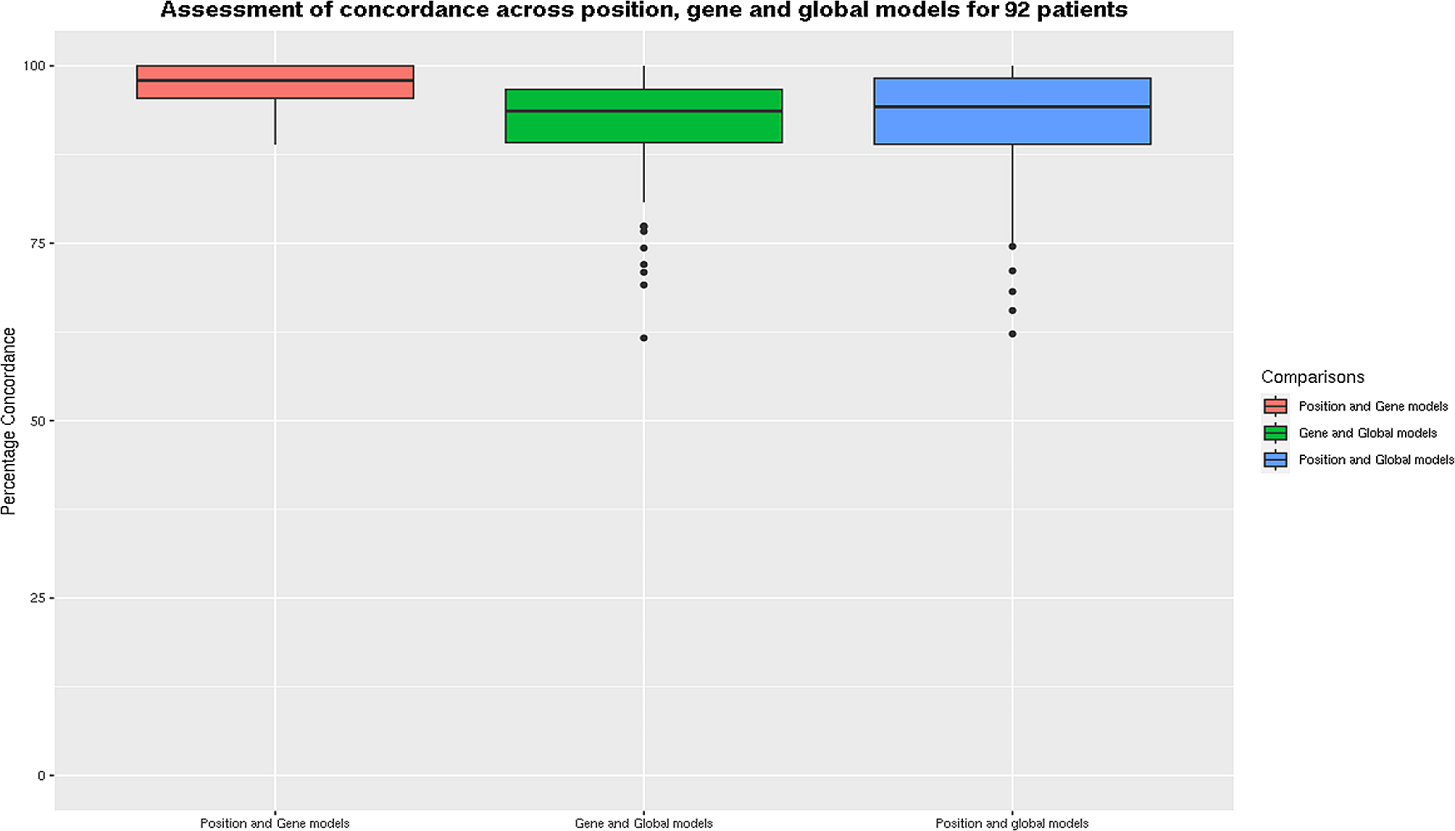
The graph shows the percentage of positions that shared the same assessment for any given position between the position and gene models, gene and global models and position and global models. The pairwise comparisons across the 3 models show > 80% agreement in assessment of significance across 92 samples

Figure 4 illustrates results of the 3 models for estimation X-skew in the 11 clinically validated samples. The results show the majority of skew estimates use the position model suggesting that the reference population provides sufficient information for querying variant positions within female patients. This is seen consistently across all 11 samples and is not biased by clinical skew measures. The percentage of positions used to assess skew are in agreement across the three models compared pairwise (position and gene models, gene and global models, position and global models) for all 92 samples in the validation and application cohorts (Fig. 5). The data shows that the position and gene models have > 95% agreement, gene and global models have > 90% agreement, and all three models (position, gene, global) have > 80% agreement for assessment of skew across the cohort. When querying a position against the reference population for assessment of outlier skew, the position and gene-based model fit a beta binomial distribution on the exact position and gene from the reference population as the patient, hence we expect to see marginally higher agreement between them. The global model uses 2000 randomly sampled positions across all coding regions of the X chromosome. The data presents evidence that the gene and global models are effective surrogates in the absence of the position-based model from the reference for estimating skewness. The analysis justifies the use of the logic behind the hierarchical use of the position, gene and global models as described in the methods section (Details in supplementary methods).

### Genes associated with variants contributing to skewed XCI

The proposed method predicted skewed patterns of XCI in 7 patients within our validation cohort (*N* = 11) and in 8 patients within our application cohort (*N* = 81). A list of impacted genes consisting of variants reported to show biased expression is provided in supplementary Table 1.

### Impact of presence of escape genes on XCI status computed using NGS data

Using literature and experimental evidence gathered from previous studies [14, 15], Katsir et al. [16] reported 38 genes with high confidence of escaping XCI (escape genes). 22 of 38 escape genes are present outside of PAR regions. We evaluated the presence of significantly skewed variants in these regions within samples predicted to show skewed patterns of XCI using NGS data in our validation (*N* = 11) and application cohorts (*N* = 81). One escape gene was found to be impacted by the presence of significantly skewed variant positions in 3 samples in our validation cohort (Supplementary Table 2). From the application cohort, only 1 escape gene was found to be associated with a significantly skewed variant position in 1 sample. (Supplementary Table 2).

Additionally, we evaluated the impact of calling X-skew on the samples above by excluding the escape genes from the computation of percentage of significantly skewed variants present in a sample. For both the validation and application cohorts, exclusion of the escape genes did not change the prediction of X-skew status (Supplementary Table 2).

### Allele specific methylation (ASM) analysis using WGBS (whole genome bisulphite sequencing)

We used WGBS to analyze the ASM in promoters including exon 1 for all genes on the X chromosome outside of PAR regions in 10 of 11 samples from our validation cohort. One sample was excluded due to technical limitations. Table 5 presents results of ASM showing the consistency of higher promoter methylation (mean = 35.1%) in samples found to be concordant with the clinical test for presence of skewed X inactivation. Similarly, samples concordant with the clinical test for random X inactivation presented significantly lower percentage of promoter methylation (mean = 3.8%). The sample with discrepant results (Sample_10) and the sample reported to be inconclusive (Sample_14) from clinical grade X-skew testing presented with moderate levels of ASM; 18.3% and 25.84% respectively (Table 5).

**Table 5:** Comparison between allele specific methylation (ASM) analysis (column 5) using WGBS and percentage of significantly skewed variants in the validation cohort (column 4)

Sample	GW	NGS	NGS_skew_PCT	ASM_PCT
Sample_1	skewed	skewed	73.04	40.29484029
Sample_2	skewed	skewed	22.49	29.29475588
Sample_3	skewed	skewed	46.31	38.02469136
Sample_5	skewed	skewed	40.8	33.16455696
Sample_7	random	random	2.35	0.829875519
Sample_8	random	random	8.2	4.781704782
Sample_9	random	random	2.94	5.527638191
Sample_11	random	random	2.6	2.017291066
Sample_10	random	skewed	18.3	7.973421927
Sample_14	UI	skewed	25.84	18.67088608

## Discussion

Skewed XCI is common in the general population, however the presence of moderate to extreme levels of skew in female individuals who are carriers of X linked disorders can be a key factor for phenotypic expression associated with the disease [1]. In order to overcome limitations posed by the current clinically acceptable standard, we developed a method for identification of XCI status independent of the methylation status of a single locus. We leveraged SNV level data from exome and transcriptome sequencing for regions expressed along the X chromosome. Our approach models XCI from whole blood samples from the GTEx [13] consortium to build a model for skewness observed in the general population. This reference model is used to determine if the extent of skew observed in patient samples presents as an outlier distribution in comparison to the reference, thereby, providing an indication for aberrant levels of XCI. The application of such an outlier-based analysis allows for sensitive detection of skew on a chromosome wide level in patients with suspected phenotypes for rare genetic disorders.

A recent study evaluated XCI in the general population and served as a precedent for understanding the levels of skew observed within healthy controls [1]. This study also extended analysis of XCI beyond the *AR* locus using sequencing, however it required the presence of parental genotypes which are not readily available in case of patients on diagnostic odysseys. To overcome this limitation, we developed a method which does not require the presence of parent samples. The data generated from our integrated analysis of ES and transcriptome sequencing data compared with a profile of normal healthy controls provides a wealth information necessary for provoking insightful investigations into disease etiology of the patients evaluated.

Herein, we describe a method for identifying skewed and random patterns of XCI using NGS data from ES and transcriptome sequencing. We built a reference model for XCI using female samples from the GTEx consortium [13]. The reference model was used for deploying an outlier analysis technique for determining X-skew in a cohort of rare disease patients. We tested the method for identifying XCI status in 11 female patients presenting phenotypes consistent with suspected rare diseases seen at Mayo Clinic for genetic testing. These patients underwent clinical grade testing for X-skew using the AR gene assay. Finally, we evaluated skewed and random patterns of XCI in additional 81 female patients presenting to the clinic with undiagnosed genetic diseases. Our method showed high concordance with the current clinical XCI test results. We note that the discordant results from 1 patient within our validation cohort (Tables 1, 2 and 3) identified as skewed using the proposed method, in contrast to the random inactivation patterns detected from the clinical assay may reflect biological changes that cannot be captured using the focal clinical testing of the *AR* gene. For these cases, the percentage of variants presenting with significantly biased expression is similar and closer in measure to the percentages seen in samples where the skew predicted from NGS data agrees with findings from the *AR* assay (Fig. 2). The assessment of skew from multiple data points along the X chromosome provides increased confidence in support of the predicted XCI pattern as opposed to one single locus. This leads us to believe that, even though the clinical assay predicts a random pattern, the presence of biased expression along the X chromosome in higher quantities compared to a healthy population, provides biological evidence for investigating the observed skew which would otherwise not be investigated due to findings of random X-skew from the *AR* gene assay.

To further support the evidence observed using our approach, we performed ASM analysis using WGBS on 10/11 samples from our testing cohort. The consistency of measures between ASM data and our NGS based method for calling X-skew validates the concordance of the epigenetic and transcriptional processes associated with XCI. The moderate levels of ASM observed in the discrepant sample (Sample_10) and the sample found to be inconclusive for clinical X-skew testing (Sample_14) provides support for evaluating XCI as a chromosome wide process shedding light in areas that maybe relevant in revealing diagnostic candidates that maybe missed owing to assessment of skew in a focal region as determined by clinical grade testing. Further, a recent study [17] conducted using long read nanopore sequencing for precise quantification of XCI using multiple methylation sites across the *AR* and *RP2* genes demonstrated the limitations of the clinical grade X-skew testing particularly in cases where XCI patterns exhibit low to moderate skewing. This is consistent with our findings and future experiments on X chromosome wide long read sequencing would be significant for revealing the complexities of the epigenetic control of XCI in patients presenting with partial skew as observed in 2 of our validation samples (Sample_10 and Sample_14).

The XCI result predicted by our method in Sample_10 is further supported by familial testing (Figs. 2 and 3; Table 3). Sample_10 had a phenotype consistent with Dubowitz syndrome, a rare autosomal recessive disorder marked by multiple congenital developmental abnormalities and is known to be a collection of phenotypically similar disorders [18]. This patient shared the phenotype with an affected maternal aunt (Sample_3) who showed skewed XCI using both the AR gene assay and our method. The mother of the patient (Sample_9) was unaffected and was reported to be randomly skewed. While investigating the connection between Dubowitz syndrome [18] and skewed XCI is beyond the scope of this study, it provokes questions on whether biased expression of genes on the X chromosome might be linked to the intrinsic etiology of the disease. Therefore, this example case highlights the potential value revealing key insights necessary for diagnosis in these challenging, ultra-rare genetic conditions. Additionally, the shared phenotypes between the proband and aunt and equivalent X-skew results obtained from NGS data agree with the CVAC from the transcriptome data for both proband and aunt (Supplementary Fig. 1). These observations present a case of possible unique progenies and inheritance patterns, an observation unusual in Mendelian disorders because, the aunt and proband reveal similarities in their profile of variants on the X chromosome as opposed to the differing skew results between parent (Sample_9) and proband (Sample_10).

Two case vignettes are used to demonstrate the clinical diagnostic utility of our approach in the realm of ultra-rare genetic disease [19]; one positive case and one negative case for skewed patterns of XCI with a suspected diagnostic endpoint. The positive case involved the identification of X-skew in a female individual with a variant of unknown significance (VUS) found using ES in the *WDR45* gene predicted to cause aberrant splicing of the canonical transcript. *WDR45* is an X-linked dominant disorder known to be disease causal through a loss of function mechanism via germline pathogenic variants often presenting with lethality in males and variable expressivity in females, possibly correlating with XCI [20–22]. RNA-seq analysis revealed the following 2 findings: (1) mis-splicing of the *WDR45* transcript with splicing occurring from exon3 to exon5 in the *WDR45* gene resulting in an in-frame deletion of exon 4, (2) Skewed XCI as determined by the presence of 44% of variants significantly skewed along coding regions of the X chromosome. The presence of an in-frame deletion with the deleterious impact of the mis-splicing in *WDR45* gene in conjunction with the identification of skewed XCI supported the elevation in classification of the variant to likely pathogenic thereby providing a genetic diagnosis. In the second case, a female patient suspected of having Fabry disease, an X-linked disorder associated with the *GLA* gene based on clinical and biochemical findings was tested for skewed X-inactivation using our method [23]. We predicted random XCI in the individual with 2.5% (2/77 SNPs with > 10 reads) of variants observed to be skewed. This finding triggered a more comprehensive genetic evaluation of the ES data which detected a pathogenic variant in the *LMX1B* gene, ultimately providing the patient with a diagnosis [24]. Based on our approach, *GLA* was deemed not related to the patient’s phenotype. This negative case study presents our method’s applicability as a tool for ruling out suspected candidate variants/genes as causative for phenotypes associated with rare diseases.

Although XCI involves silencing of either parental copy, nearly 15% of X-linked genes are expressed from both the active and inactive X-chromosomes. Genes known to escape XCI present variable degrees of XCI escape between genes, tissues, developmental phase and individuals [1]. Owing to the sparse nature of variant calls, it is important to evaluate the impact of the presence of escape genes on the accuracy of our method. Our method relies on the availability of high confidence heterozygous variant allele counts from RNA sequencing data. To account for the possibility of a false prediction of random XCI in a skewed sample, owing to the presence of lesser number of significantly skewed variants in proportion to the number of biallelic variants expressed from the escape regions, we computed the percentage of significantly skewed variants while excluding the escape genes. In doing so, we noticed no change in the predicted outcome for samples predicted to be skewed in our validation and application cohorts. Supplementary Table 2 lists the percentage of significantly skewed variants computed with and without including escape genes for all samples in the validation and application cohort respectively that consisted of significantly skewed variants in genes confirmed to escape XCI. The analysis supports the robustness of the method in determining patterns of XCI independent of the presence of genes that escape XCI or show variability in escape from XCI.

RNA editing may also bias the results of the X-chromosome inactivation predictions generated in this study. The most common type of RNA editing that occurs in the human genome is the A-to-I (Adenosine to Inosine) editing mediated by the ADAR enzyme, occurring predominantly in the 3’ UTRs and intronic gene regions [25, 26]. RNA editing mediated monoallelic expression may result if: (1) an intronic RNA edited base leads to altered splicing and the creation of an out-of-frame product from one allele, subsequently causing an SNV on the other second allele to appear homozygous; (2) an RNA edited base in the 3’UTR. Both events are site specific and focal to the gene. As this method identifies skewed X inactivation using multiple sites across the coding regions of the X chromosome (with the exception of PAR regions), we reason, the odds that multiple sites included in XCI prediction are impacted by RNA editing would be very low. Thus, we do not anticipate that RNA editing events would significantly impact our prediction of X-skew. To assess this, we studied known RNA editing sites catalogued in human blood to determine if they occurred within sites considered in our study assessments. From the 43,235 RNA editing sites catalogued within the REDIportal on the X chromosome in whole blood tissues [27], we observed two sites that occurred in our predictions. TCEAL4 (exon 1) and XIAP (exon 6) each contained one position, which was used in our assessment of X-skew across 11 samples, 2 of which were predicted to be skewed. However, the significance score for both loci did not pass our threshold of *P* < 0.05 and thus did not significantly impact the percentage of positions found to be significantly skewed any of the samples tested. Additionally, we reviewed RNA editing events catalogued in two studies: one focused on coding RNA editing events in pediatric cancer and the other assessing the clinical relevance of A-I editing in human malignancies [28, 29]. None of the RNA editing sites documented in these studies overlapped with the loci used for assessment of X-skew in our evaluation and validation cohorts. This analysis supports the notion that although RNA editing is a prevalent post transcriptional mechanism in whole blood, it does not significantly impact the X-skew predictions made in this study.

Multiple studies have reported the use of transcriptome sequencing in combination with genotyped data for inferring allele specific expression using an outlier-based analysis method in rare disease cohorts [12, 30]. However, the rare disease cohorts used were limited to muscle disorders and the prime focus of these studies was geared towards mining candidate genes associated with muscle disorders [12, 30]. Previous studies on evaluation of XCI status in the females using RNA sequencing have demonstrated that patterns of XCI observed in whole blood are consistent with XCI status at the embryonic stage and across tissues [1, 6]. Since blood is easily accessible and readily available as a tissue source, we reason that the results from the proposed study can be investigated for the observed phenotypes in inherited X linked disorders and the results can be at least partially indicative of skewing in other relevant tissues of interest. One study utilized patient samples from 16 different disease types and used controls from whole blood for assessment of allelic variation, however, the study used only transcriptome sequencing for determining allele specific expression within genes and sites that were common to patient and control samples [11]. One of the advantages of the method used in our study over previously published studies is that, for observations within a patient sample that may not be represented within the GTEx cohort, a global model was applied that estimates if the patient site shows biased expression by drawing a beta binomial distribution using random sample of positions within the reference cohort. The presence of such a model provides flexibility for mining skewed expression within loci that do not have representation within the reference cohorts, thereby fully extending the utility of our approach to all coding regions outside of PAR regions within the X chromosome. Finally, the availability of chromosome wide data for evaluating XCI status not only allows for disease diagnosis but also serves as a means of an effective genetic screening tool for newborn females by allowing assessment of disease risk for carrying variants with deleterious X-linked variants [6].

Although the reported work detects skewed and random XCI patterns, it is important to address technical variables that might impact the performance of the method. We recognize the differences in library preparation methods used for the GTEx and rare disease cohorts (See methods). To assess the impact of the library differences, we analyzed our combined validation (*N* = 11) and application cohort (*N* = 81) of patient samples for XCI status against a model generated using the same patient cohort of 92 samples (Supplementary Fig. 2). We observed a similar threshold of 12% aligned to a local minimum and provided a reasonable discrimination point for called skewed X-inactivation, suggesting transferability of the method between study cohorts. Supplementary Fig. 3 presents the distribution of samples from the application cohort (*N* = 81) modelled against the normal GTEX reference cohort (See methods). Given the difference in cohort parameters (library preparation methods, sequencing sites, etc.) an adjustment of threshold from 14% (Fig. 1) to a threshold of 12% (Supplementary Figs. 2, 3) was made to align to a local minimum in the overall cohort distribution. It is suggested that users implementing this method on a unique cohort validate the distribution of computed values and adjust the threshold to a local minimum reflected in the study to align with an expected skew identification rate of 10–20% in the population. In both the analyses (Supplementary Figs. 2,3), the results of XCI status presented consistent outputs for samples that were identified to present skewed and random XCI patterns. The threshold of 12% used in this study aligns with previously reported estimates of the frequency of X-skew observed in the population [1, 6] and was derived using a local minimums on the density plots (Fig. 1, Supplementary Fig. 3) [1, 6]. Although our applied threshold represents marked differences in the number of significantly skewed variant positions between samples predicted to be show skewed and random XCI in the validation cohort (Table 1), we realize the potential presence of thresholding effects in cases that are on the lower bound of the range of the percentage of significantly skewed variants in our application cohort (Table 4). It is worth noting that such observations could potentially indicate the varying degree of skewness observed in the underlying samples not only at a cellular level but also along the X chromosome. Careful assessment of X-linked genes impacted by the presence of such variants using integration of RNA-seq and methylation approaches, phenotypes available and associated clinical metadata would be recommended when guiding diagnostic processes. Another constraint of our study is the limited utility of whole blood as a sample type that may not always capture the complete spectrum of XCI patterns across different tissues in all instances of rare diseases. Expanding the analysis to include other tissue types can enrich the understanding of skewed XCI patterns as shown by Tukianen et al. [15]. Our study provides a foundational first step towards more in-depth research and investigation of XCI in a clinical setting.

Our method relies on building position, gene and global models using data from the reference population. We observed that parameter estimates calculated for position specific, gene specific and global models did not present any significant regional bias and GC content bias (Data not shown). These observations provide evidence in support of the robustness and reproducibility of the models thereby emphasizing the relevance of using an approach that relies on the beta binomial distribution. One of the limitations of our method is that it relies on the presence of expressed heterozygous SNVs from transcriptome sequencing with sufficient depth of coverage. Since such positions are relatively sparse along the X chromosome, the exclusion of multiple regions from a sample could potentially reduce the data available for assessment of X-skew.

## Conclusions

The field of translational medicine has been revolutionized by advances in next-generation sequencing technologies which are leading to increased diagnostic yield in patients with rare genetic disorders [31, 32]. Complex biological processes can be probed by integrating information from DNA and RNA sequencing (RNA-seq) methods. Rare genetic diagnosis informs the presence of abnormal events at various molecular levels including the genome and transcriptome. Currently diagnosis of Mendelian disorders remains a “needle-in-the-hay-stack” problem due to the rare occurrence of different types of genetic diseases making them challenging to investigate and characterize, owing to a limited number of affected individuals for each type of rare disorder. One of the ways to identify abnormal events is by distinguishing events at the molecular level in a given patient in comparison to a normal healthy population. Herein, we present a method for evaluation of XCI status within a cohort of female patients harboring symptoms consistent with the presence of different types of rare genetic diseases. Our approach leverages the presence of GTEx females as healthy controls for modelling XCI variation within the general population and provides a comprehensive view of XCI status along the coding regions of the X chromosome. In agreement with surveyed literature, we observed 10–14% of females show skewed patterns of XCI [1, 6]. We tested our approach on 11 female patients undiagnosed for rare germline disorders and demonstrate comparable results for our method with the available clinical test. Additionally, ASM analysis using WGS provided comparable levels of ASM measures with our X-skew results suggesting the robustness of our approach in support for observed levels of skew samples inconclusive for clinical X-skew testing. The technical limitations of the clinical assay make the proposed work more reliable for assessment of XCI status. The concordance of results with the current clinical grade testing for XCI, comparable measures of ASM and findings consistent with previously reported literature provide evidence for the clinical potential of this method for detection of XCI status in female patients on a diagnostic odyssey for inherited rare disorders. The proposed work makes use of existing ES and RNA-seq data available within the diagnostic odyssey clinic owing to the familial hereditary nature of genetic disease testing in individuals with rare genetic disorders. It is worth noting that, it can be optimized using a high throughput targeted RNA-seq assay of the X chromosome to off-set the sequencing costs from ES and RNA-seq. Finally, although the outlier-based method used in this study is implemented on females presenting to the clinic with possible rare inherited disorders, its utility for determining X-skew can be extended to other common X-linked disorders.

## Methods

### GTEx: reference model data

Sequencing data from whole blood for exome and transcriptome sequencing was downloaded from the GTEx consortium V7 [13] for 135 female samples. Sequencing data was reanalyzed internally using the same bioinformatics pipelines described below (TREAT and MAP-Rseq).

### Female individuals: validation and cohort study sample set

The female individuals used in this study comprised of 11 patients used for validation and an additional 81 patients used for X-skew evaluation. All patients presented to the Mayo Clinic Department of Clinical Genomics with phenotypes consistent with the presence of rare genetic diseases as previously described [19]. The patients included in this manuscript were part of an ongoing research study on rare and undiagnosed disease, with partial but not complete concordance with the patients previously described [19]. Samples were analyzed with exome and transcriptome sequencing for detection of causal events such as rare variants, aberrant expression, and aberrant splicing within candidate genes of interest as curated by the team of consulting physicians and medical experts. Clinical or research grade ES was done for all patients as described previously [19]. RNA from whole blood for all patients was extracted in a PAXgene Tube following manufacturer’s instructions (Qiagen). The miRNeasy Mini kit from Qiagen was used for isolation of RNA and 101 bp paired libraries were prepared using capture probes from the TruSeq® RNA Access Library Prep Kit (Illumina). Samples were sequenced at Mayo Clinic Medical Genome Facility (MGF) using Illumina Hiseq 4000 generating on average greater than 100 million total reads per sample with an estimated library size greater than 10 million reads to avoid PCR bias resulting from the library preparation protocol. Paired end libraries for WGBS were prepared using 100ng genomic DNA according to the manufacturer’s instructions for NEBNext Enzymatic Methyl-seq (EM-seq) ) (New England BioLabs, Ipswich, MA). The concentration and size distribution of the completed libraries are determined using the Fragment Analyzer (Agilent, Santa Clara, CA) and Qubit fluorometry (Invitrogen, Carlsbad, CA). Libraries are sequenced as 150 × 2 paired end reads using NovaSeq S4 (Illumina, San Diego, CA) sequencing kit and NovaSeq Control Software v1.8.0. Base-calling is performed using Illumina’s RTA version 3.4.4 resulting in over 1 billion per sample.

#### Bioinformatics

For all ES data used in this study, the fastq files for paired end 101 bp reads were aligned to human genome reference build hg19 using BWA version 0.7.10. Variants were called using GATK haplotype caller [33]. FASTQC was used for the assessment of quality control metrics. All data were processed using a bioinformatics pipeline developed internally at Mayo Clinic called TREAT [34].

For all transcriptome sequencing data used in this study, the MAP-Rseq [35] pipeline was used to process the RNA sequencing reads, aligning them against human genome and transcriptome for reference build hg19 using Tophat2 [36]. Quality control of transcriptome sequencing data was done using RSEQC [37].

For WGBS data, sequenced reads were aligned to human genome reference build hg19 using BS-Seeker2 [38]. CGMAPTOOLS [39] was used to generate ATCG map files and SNVs were called using the bayes mode with the following options (-m bayes–bayes-dynamicP). Alelle specifically methylated (ASM) sites were identified using the ass mode in CGMAPTOOLS with the following options -m ass -d 10 -L 0.4 -H 0.6. A bed file was prepared using promoter regions for all coding regions on the X chromosome outside of PAR regions including exon 1 of every gene [40] using hg19 annotations for promoter sequences from Ensembl [41]. The percentage of significant ASM sites (*P* < 0.05 and ASM = TRUE) for each sample was calculated as a fraction of the number of sites evaluated across all promoter regions.

### Estimation of allele counts using ES and transcriptome sequencing for patient samples and GTEx controls

**Fig. 6:**
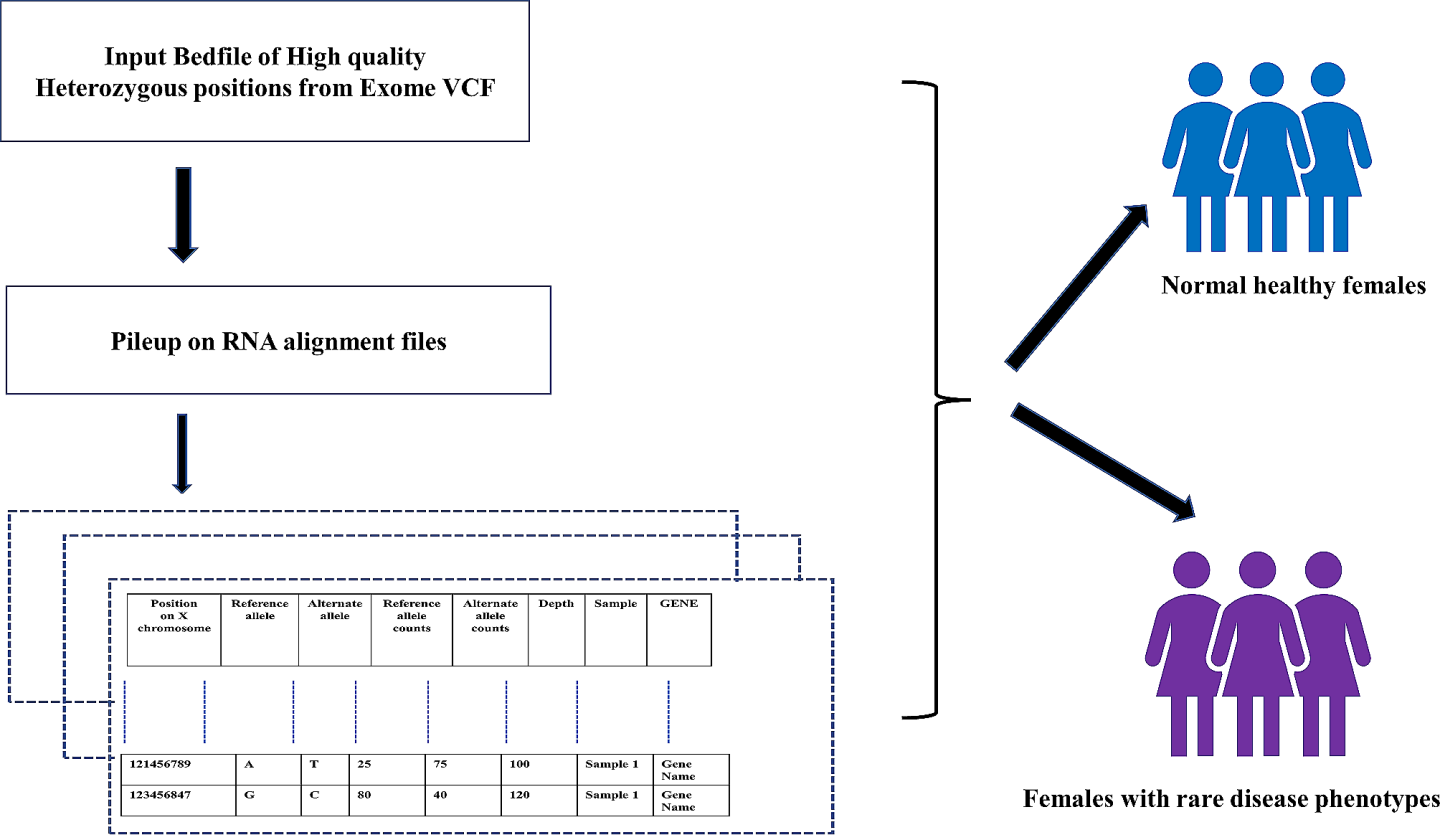
Workflow for extracting CVAC for transcriptome sequencing guided by heterozygous genotypes from Exome sequencing data

This work uses single nucleotide variants (SNVs) and 1 bp indels from coding regions of the genome as annotated from the RefSeq gene track from the UCSC Browser [42] for detecting the presence or absence of skewed inactivation along the X chromosome. We rely on high-quality heterozygous positions present within the exome sequencing data and mine the corresponding transcriptome allelic counts for both reference and alternate alleles. We restricted our analysis to exclude the pseudoautosomal regions (PAR). Figure 6 illustrates the workflow for extracting allelic counts from RNA sequencing guided by high confidence heterozygous genotypes from ES. The following steps were used for female controls from GTEx and female patients in our rare disease cohort. (Details provided in supplementary methods, part 1)

(i) For each sample, the exome variant calls were subsetted to include heterozygous variants outside of PAR regions on the X chromosome using the Select Variant tools from GATK [33].
(ii) All positions that failed the Variant Quality Score Recalibration (VQSR) were filtered.
(iii) Positions covered by > 10 reads and with a genotype quality of greater than or equal to 20 were used to create a bedfile for querying the alignments from RNA sequencing reads.
(iv) Mpileup from Samtools [43] was used to generate pileup files from RNA sequenced reads filtering all reads with mapping quality less than 20 and base quality less than 30 for all high confidence heterozygous positions.
(v) Custom python scripts were used to parse the mpileup files resulting in tab-delimited text files consisting of nucleotide information on the position of the SNV, the reference and alternate alleles, and their respective counts or the number of reads covering both the alleles.

Hereafter, we refer to the variant allele counts calculated in step (v) as the CVAC (computed variant allele counts). Additionally, for each position, gene-based annotations are added to the CVAC files using the RefSeq gene track form the UCSC browser [42].

### Using CVAC files from GTEx females for generating models representing XCI in the normal population– position-specific, gene-specific, and global models

We used data from 135 females from GTEx to build a model that represents XCI in the normal population. Reference allele count (R_i_) and alternative allele count (A_i_) were computed for all variant positions ‘i’ across ‘S’ samples and denoted as: R_i_ = {r_1i_, r_2i,_…,r_Si_} and A_i_={a_1i_,a_2i,_…,a_Si_}, where r_ji_ and a_ji_ are the reference and alternative CVACs at the i^th^ genomic position in the j^th^ sample. If each individual in the cohort had identical probability of expressing the reference versus alternative allele, a binomial distribution could be used to model the R_i_ and A_i_ counts at the i^th^ position. However, this assumption of equal probability across the samples will not hold since each female is expected to have varying degrees of skew at each position. Therefore, we model each female’s true-but unknown probability of expressing the alternate allele as being drawn from a beta distribution. After integrating out this latent probability, our resulting model is known as a beta-binomial distribution, which accounts for the overdispersion in our data compared to a standard binomial model.

To achieve this model, we used the GAMLSS [44] package in R to fit a beta-binomial probability distribution on the counts of reference and alternate alleles in ‘S’ GTEx samples for a given variant position (Supplementary Methods). Counts generated from sequencing alignments can often have very small values or absence of coverage resulting in many positions with 0 counts. We therefore followed other recent beta-binomial outlier approaches [45] and applied the regularization method of Laplace smoothing to our CVAC prior to fitting the beta-binomial model. The resulting parameter estimates are the mean $\widehat{{\mu }_{i}}$ and dispersion $\widehat{{\sigma }_{i}}$ for the i^th^ position. These parameter estimates capture the biological and statistical sampling variability of the reference and alternate allele counts within the normal population for any given position i.

This process is repeated for all positions within the GTEx cohort of 135 females. 11,382 variants were covered by 3 or more reads across the GTEx female cohort, resulting in 1725 unique variant positions being included. Our criteria for fitting a beta binomial distribution for a given position included applying a filter of 3 or more reads covering a position that is observed in 10 or more samples. We define the model generated by fitting a beta binomial distribution for variants within the GTEx cohort as the *position specific model* (See details in supplementary methods, part 1).

Similarly, we used the GAMLSS package to fit a beta-binomial distribution for allelic counts from RNA sequencing data for heterozygous variant positions in each gene within the GTEx cohort. We used the same criteria for fitting a beta binomial distribution for a gene as those described above for the position specific model (See details in supplementary methods, part 1). The beta-binomial probability distribution was used to derive parameter estimates for a given gene. Using this method, we derived a probability distribution for allele counts in 171 genes within the GTEx cohort. We define such a model as the *gene specific model*.

Both the position and gene specific models described above represent only variant positions in the GTEx samples that have at least 10 observations used for fitting a beta-binomial distribution. To model counts of variant positions not included in the position and gene specific models, we randomly sampled 2000 variant positions from a total of 11,382 positions within the GTEx cohort. We used the GAMLSS package [44] to derive a beta-binomial probability distribution that represents and captures the biological and statistical sampling variability of the reference and alternate allele counts across the 2000 positions. We define this as *the global model*.

### Outlier analysis based approach for estimating likelihood of skewed X inactivation

**Fig. 7:**
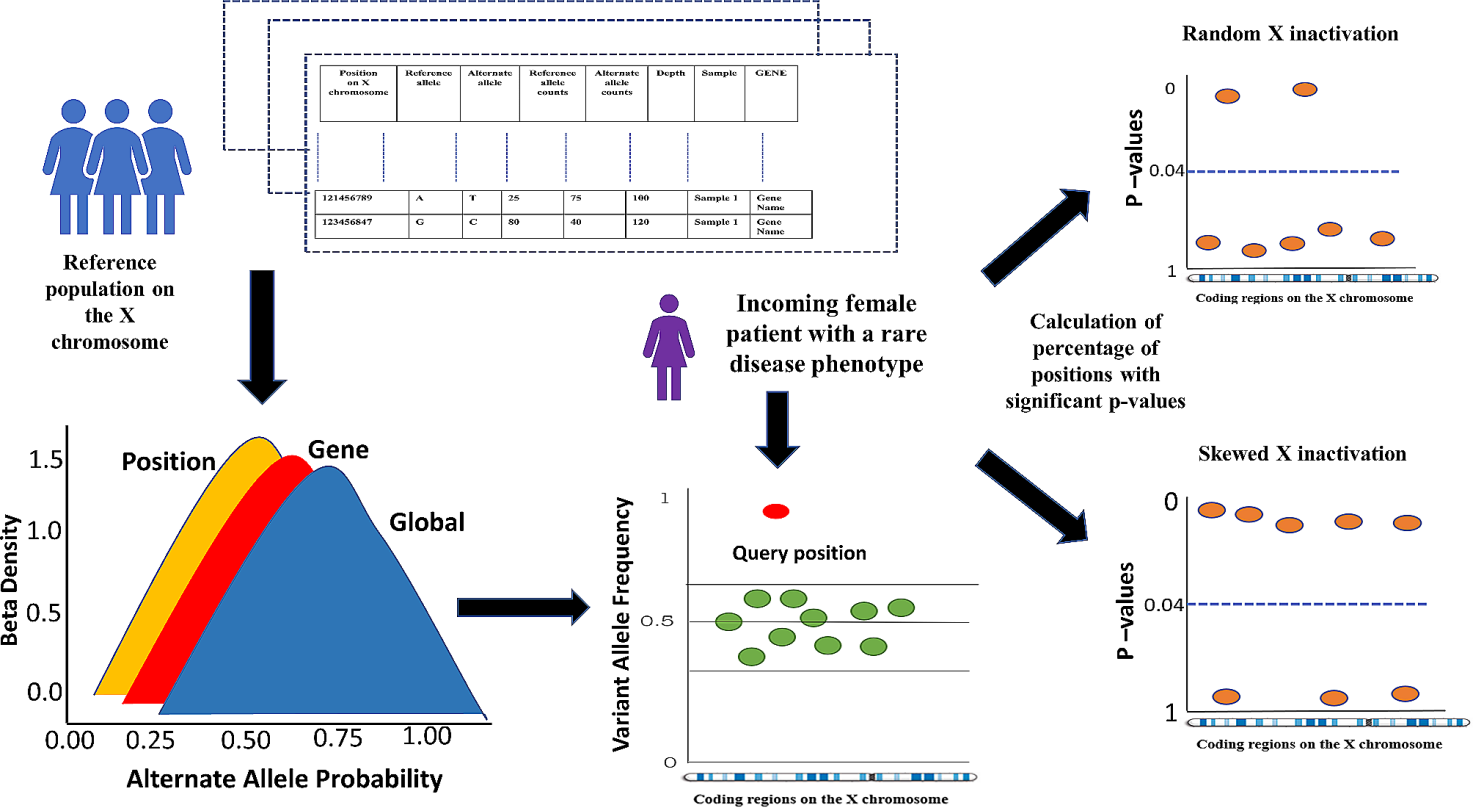
Schematic representation of outlier based analysis on rare disease females by modelling XCI data from the normal population on the X chromosome

We utilized the three models to determine positions in each sample that deviated statistically significantly from the healthy population in GTEx. Our null hypothesis for the position specific model states that the reference and alternate allele counts at that position were drawn from the beta-binomial model fit to the normal female population; rejection of this null hypothesis would indicate an outlier from the healthy population’s distribution. Similarly, the null hypothesis for the gene model states that for a female patient sample at a given position in the gene, the CVAC for that patient are distributed according to the beta-binomial gene model within the normal population. Finally, the global model has a null hypothesis that CVAC from the patient sample at this position were drawn from the beta-binomial model fit on the X chromosome to the GTEx cohort. We compute outlier *P*-values as described previously [45] for all positions with depth of coverage greater than 10 in the patient, and reject the null hypotheses if the two-sided *p*-value test is less than 0.05, indicating that the CVAC in the patient sample are significantly different than the distribution of the normal population. To identify statistically significant outliers on the X chromosome for each patient, we preferentially used first the position model score, then the gene model score, then the global model score, based on score availability. Figure 7 shows a schematic representation of the outlier-based analysis approach described above using the position, gene and global models on rare disease females by modelling XCI data from the normal population on the X chromosome (Details in supplementary methods, part 2). In this way for any given sample, all high confidence heterozygous positions outside of the PAR regions on the X chromosome are evaluated for presence X-skew. Finally, using the total number of variant positions evaluated for X-skew in any sample, the percentage of significantly skewed variant positions is computed.

### Clinical XCI testing

The fourteen female patients with suspected X linked genetic disorders used for validation of the proposed method were clinically tested for skewed X inactivation at Greenwood Genetic Center (GGC), a College of American Pathology (CAP) and College of Laboratory Improvements and Amendments (CLIA) certified molecular diagnostic laboratory. Determination of skewed patterns of XCI in tested samples used the methylation sensitive restriction enzyme Hpall which cleaves only unmethylated sites on the polymorphic “CAG” repeats within exon 1 of the *AR* gene. PCR analysis of the CAG repeats was used to determine the status of X inactivation. The assay involves isolation of genomic DNA samples from the tissue of interest followed by digestion with methylation-sensitive restriction endonuclease Hpall. Consequently, the unmethylated allele from the active X chromosome in the tissue is digested. Digested products are PCR amplified and separated by gel electrophoresis. The presence of two bands on the gel of differing sizes represents the presence of an active X chromosome from maternal and paternal alleles. Finally, the different sizes of the parental peaks on the gel are quantified and the XCI ratio is calculated representing the proportion of cells having an active X chromosome from each parent [46].

The test reports XCI ratios of greater than 90:10 to be highly skewed suggesting strong preferential expression of one parental allele over the other in more than 90% of cells and proposes that the observed skewed XCI maybe of clinical significance to the patient phenotype. XCI ratios between 80:20 and 90:10 are reported to be moderately skewed. XCI ratios of less than 80:20 are considered to be random patters indicating normal unbiased expression of both parental alleles in cells from whole blood.

### Electronic supplementary material

Below is the link to the electronic supplementary material.

Supplementary Methods.

Supplementary Table 1: List of samples from the validation and application cohorts with age, percentage of significantly skewed variants, predicted X-skew status, number of variants evaluated using the position, gene and global models and list of genes in patients predicted to be skewed.

Supplementary Table 2: Samples from the validation and application cohorts that were predicted to be skewed using NGS data with and without the inclusion of escape genes consisting of significantly skewed variant positions.

Supplementary Figure 1: Computed variant allele frequency (Y-axis) for heterozygous variants shared between the proband (Sample_10) and maternal aunt (Sample_3) on the X chromosome. The variant frequencies from the maternal aunt and proband observed in the transcriptome are indicative of biased allelic expression and show a similar trend in both samples.

Supplementary Figure 2: Density plot for 92 individuals from the validation and application cohort for the percentage of significant p-values when tested on a per sample basis for each of the 92 females against the same patient cohort.

Supplementary Figure 3: Density plot for 81 individuals from the application cohort for the percentage of significant p-values when tested on a per sample basis for each of the 81 females against the GTEx reference cohort.

## Data Availability

The datasets used and analyzed in the current study are available in the GEO repository https://www.ncbi.nlm.nih.gov/geo/query/acc.cgi?acc=GSE234607 under the accession number GSE234607. Custom Scripts for generation of position, gene and global models and step-by-step instructions are provided at https://github.com/nmfad/X-chromosome-analysis.

## Abbreviations

CVAC: Computed Variant Allele Counts
DMD: Duchenne Muscular Dystrophy
GTEx: Genotype Tissue Expression Consortium
PCR: Polymerase Chain Reaction
VQSR: Variant Quality Score Recalibration
XCI: X chromosome inactivation
