# Supplementary material for: Identification of skewed X chromosome inactivation using exome and transcriptome sequencing in patients with suspected rare genetic disease
Source: BMC Genomics. 2024 Apr 16;25:371. doi: 10.1186/s12864-024-10240-2 (PMC11020449; doi:10.1186/s12864-024-10240-2)
Supplement: Supplementary file 1 — Supplementary Methods. [file 12864_2024_10240_MOESM1_ESM.docx]

Supplementary Material

# Supplementary Methods

**Analysis of X chromosome inactivation in female patients with rare gene genetic disorders.**

The method outlined below assumes that VCF files are available from exome sequencing and alignment files are available from RNA sequencing (See Materials and methods in the main text for the bioinformatics pipelines used for generating the VCF files and bam files using human reference genome build hg19. Note: Users can use any bioinformatics pipeline of choice, however if using different alignment and variant calling methods than the ones incorporated in the pipelines referenced, caution must be exercised when filtering out false positive variant calls from DNA VCF files).

**Part 1: Building a beta-binomial model using healthy control female population from the Genotype Tissue Expression (GTEx).**

Step 1: Paired end exome sequencing (ES) fastq files for 135 GTEx females were processed using the TREAT bioinformatics workflow^16^. Alignment was done using BWA version 0.7.10. and variants were called using GATK haplotype caller.

Step 2: For each sample, exome variant calls were subsetted to include only heterozygous SNVs and 1 bp indels on the X chromosome. Variants with a genotype of 0/1 in the VCF were selected as heterozygous variants and filtered to include only heterozygous positions that

1. Were annotated as “PASS” by the Variant Quality Sore Recalibration (VQSR) module from GATK
2. Were present outside of PAR (pseudoautosomal) regions. A bed file for all regions of the X chromosome outside of the pseudoautosomal regions is located at <https://github.com/nmfad/X-chromosome-analysis>
3. Had a depth greater than or equal to 10 total reads (DP >=10) and had a genotype quality (GQ >20)
4. And present only within exon regions on the X chromosome. A bed file consisting of only coding regions on the X chromosome is located at <https://github.com/nmfad/X-chromosome-analysis>

Step 3: The resulting VCF file obtained from step 2 was converted to a sorted bed file. Steps 1 – 3 result in the generation of 135 bed files corresponding to high quality heterozygous variant positions on all coding regions of the X chromosome excluding PAR regions.

Step 4: Paired end RNA sequencing files for 135 GTEx females were processed using the MAP-Rseq^17^ pipeline. (See materials and methods in the main text)

Step 5: For every sample, samtools mpileup was used to compute an mpileup file using (a) the RNA sequencing bam file generated in step 4 and (b) The bedfile for high quality heterozygous positions generated in steps 1-3. A minimum mapping quality threshold (-q) of 20 was used and a minimum base quality of (-Q) of 30 was used. An example command for running samtools mpileup using the RNA sequencing bam file and exome based bed file discussed earlier is provided within the readme file located at <https://github.com/nmfad/X-chromosome-analysis>. This step results in the generation of mpileup files for every sample consisting of pileup information for each heterozygous variant position obtained in steps 1-3.

Step 6: Using the mpileup files from step 5, the next step is to calculate the computed variant allele counts (CVAC) within the RNA data (See main text in Materials and Methods). A custom python script was written to parse the mpileup files generated in step 5 resulting in tab delimited files consisting of reference and alternate allele counts in the RNA data corresponding to the heterozygous positions derived from steps 1-3. The tab delimited CVAC file consists of the following columns: chromosome, position, reference allele, alternate allele, reference allele count, alternate allele count, depth, alternate allele frequency, reference allele frequency and sample name. The custom python script for parsing mpileup files from RNA using bed files from step 1-3 is located at <https://github.com/nmfad/X-chromosome-analysis>. The script is run on every sample resulting in 135 CVAC files.

Step 7: The next step is to annotate the positions in the CVAC file for gene information using the gene bed file for all coding regions on the X chromosome. The step uses the bedtools intersect command on the CVAC file generated in step 6 and the bed file mentioned in step 2(d).

Step 8: In this step, the 135 CVAC files computed in step 7 are combined together to create a large file which will be used as input for creating the gene, position and global models (See Material and Methods in the main text). Additionally, the resulting file is also filtered down to include only positions that are covered with a total depth of over 3 reads. This is done to avoid providing the model building step with positions of negligible coverage.

Position Model: For every position observed 10 or more times in the GTEx (healthy control) population, the position model fits a beta binomial distribution using reference and alternate allele counts (CVAC calculated in step 6). The model estimates parameters called muHat and SigmaHat for a given position. We require that a variant position is observed at least 10 times in the healthy GTEx controls to be able to provide the model with sufficient information for estimating the parameters (muHat and SigmaHat). This resulted in the generation of parameter estimates for 1725 variant positions. The estimates are output in a tab delimited file consisting of the position, muHat and SigmaHat for 1725 positions. To achieve this model, the GAMLSS^22^ package in R was used to fit a beta-binomial probability distribution on the counts of reference and alternate alleles in GTEx samples for a given variant position. The scripts and example files for parameter estimation for a position specific model can be found on <https://github.com/nmfad/X-chromosome-analysis>

##### Gene Model: For any gene that consists of a total of 10 or more variants across the GTEx population (i.e if there are 2 variants in gene A in a GTEx sample 1 , 3 variants in gene A in GTEx sample 2 and 5 variants in gene A in GTEx sample 3, there are a total of 10 variants present for gene A within the control population), the gene model fits a beta binomial distribution using reference and alternate allele counts (CVAC calculated in step 6). Similar to the position model, for every gene (that consists of 10 more total variants) parameter estimates SigmaHat and muHat are calculated. Using this method, a probability distribution for 171 genes was derived using variants present in the GTEx population. The scripts and example files for parameter estimation for a position specific model can be found on <https://github.com/nmfad/X-chromosome-analysis>

Global Model: Both the position and gene specific models described above represent only variant positions in the GTEx samples that have at least 10 observations used for fitting a beta-binomial distribution. However, in order to generate a beta binomial probability distribution for variants on the coding regions of the X chromosome not represented by the position and gene models, we randomly sampled 2000 variant positions from a total of 11,382 positions within the GTEx cohort.

For ease of convenience and reproducibility, we provide the parameter estimates generated using 135 GTEx females for the position, gene and global models at <https://github.com/nmfad/X-chromosome-analysis>

Note: A variant position that occurs 10 or more times in the GTEx population consists of estimates for all 3 models: position, gene and global models. The parameter estimates (muHat and sigHat) calculated for the position, gene and global models represent the binomial probability distributions for variants on the X chromosome in the GTEx population. For any incoming test sample enrolled into the clinic for rare diagnostic odysseys, the identification of XCI patterns involves an outlier-based analysis approach using the probability distributions computed in the steps above as a reference. To determine if variants in the patient sample deviate significantly from the healthy population (See Materials and Methods in main text), probability values (P-values) will be computed for each variant in the patient sample.

**Part 2: Outlier based analysis approach for evaluating skewed patterns of XCI in 92 female individuals with different types of undiagnosed rare genetic disorders.**

Step 9: Steps 1- 7 were repeated for generating CVAC files for the 92 (patient samples with different types of undiagnosed rare genetic disorders) samples. These samples included 11 samples from our validation cohort and 81 samples from our application cohort making it a total set of N=92 patient samples.

Step 10: In the next step, the CVAC files for test samples from step 9 were used as input to query variants from test samples and evaluate if the allele counts for a given variant position deviate significantly from the allele counts for that position in the reference population (GTEx). This was achieved by computing P-values for a given position against the position-based model using the beta binomial distribution generated from step 8. If the given position does not have parameter estimates computed from the GTEx samples, then the method determines if the gene in which the variant occurs consists of parameter estimates for it from the GTEx population. If neither position nor gene level parameter estimates exist for a variant, the global model is evaluated to determine if the variant presents a significant deviation from the distribution in the GTEx samples. In this manner, using the CVAC files for 92 individuals, P-values were computed for each variant in a given test sample to determine if the variant allele counts present skewed expression in comparison to a normal reference. Script for computing P-values using the method described above is located at <https://github.com/nmfad/X-chromosome-analysis>

Step 11: For every test sample, the output from step 10 consists of a tab delimited file consisting of the following columns:

1. Position of the variant on the X chromosome
2. muHat value for the position-based model for that position (Note: It is reported as NA if the variation position is not present within the GTEx cohort)
3. SigmaHat value for the position-based model for that position (Note: It is reported as NA if the variation position is not present within the GTEx cohort)
4. P-value from the position-based model (Note: It is reported as NA if the variat position is not present within the GTEx cohort)
5. Direction of significance (increase/decrease) : If the observed skew is in favor of the alternate allele (increase) or not (decrease) based on the reported p-value.
6. muHat value for the gene-based model for that position (Note: It is reported as NA if the gene is not present within the GTEx cohort)
7. SigmaHat value for the gene-based model for that position (Note: It is reported as NA if the gene is not present within the GTEx cohort)
8. P-value from the gene-based model (Note: It is reported as NA if the gene is not present within the GTEx cohort)
9. Direction of significance (increase/decrease): If the observed skew is in favor of the alternate allele (increase) or not (decrease) based on the reported p-value.
10. muHat value for the global model (Note: this value will be the same as there is only one muHat value computed using 2000 randomly sampled variants from the GTEx cohort)
11. SigmaHat value for the global model (Note: this value will be the same as there is only one SigmaHat value computed using 2000 randomly sampled variants from the GTEx cohort)
12. P-value from the global model
13. Direction of significance (increase/decrease): If the observed skew is in favor of the alternate allele (increase) or not (decrease) based on the reported p-value.
14. Reference allele count
15. Alternate allele count
16. Gene Name

Step 12: In this step, the percentage of variants reported to be significant skewed in a test sample was calculated using the following steps.

1. Only variant positions with a depth of 10 or more reads (reference and alternate allele counts greater than equal to 10) were used towards this calculation. In the output file from step 11, the sum of columns 14 and 15 should be greater than or equal to 10.
2. The numerator in the equation below is the number of variant positions with a p-value < 0.05 and consisting of greater than equal to a depth of over 10 reads from step 11. If a position consists of a p-value from the position, gene and global models, the position based pvalue is prioritized over the gene and global models. In the absence of p-value from the position based model, the gene model based p-value is used and finally, if neither position nor the global model consists of a p-value, then the p-value scpre provided by the global model is used towards calculating the percentage of statistically skewed positions.
3. The denominator is the equation below is the number of variant positions in the test sample consisting of greater than or equal to a depth of over 10 reads from step 11.

$$Percentage of variants reported to be significantly skewed=\frac{Number of variants reported to be significantly skewed}{Total number of variants on the chromosome X in the sample}$$

1. The threshold used for identifying a sample to show skewed patterns of XCI is 12%. For any test sample, if the percentage of variants calculated from step 12 is greater than 12%, the sample was identified to show skewed patterns of XCI.

The percentage of significantly skewed positions (P-value < 0.05) were calculated for each of the 92 samples. If the percentage of significantly skewed positions less than 12%, then the sample was identified to show random patterns of XCI. If more then 12% of variant positions in the sample were observed to be significantly skewed, the sample was identified to show skewed patterns of XCI. 15/92 samples were reported to show skewed patterns of XCI when compared to the GTEx samples (Table 1 from main text). Supplementary table 1 provides a the list of samples identified to show skewed patterns of XCI and the list of corresponding genes consisting of variants with allele counts significantly skewed towards either parental allele.

# Supplementary Figures

Supplementary Figure 1: Computed variant allele frequency (Y-axis) for heterozygous variants shared between the proband (Sample_10) and maternal aunt (Sample_3) on the X chromosome. The variant frequencies for the maternal aunt and proband observed in the transcriptome are indicative of biased allelic expression and show a similar trend in both samples.

Supplementary Figure 2: Density plot for 92 individuals from the validation and application cohort for the percentage of significant p-values when tested on a per sample basis for each of the 92 females against the same patient cohort.

Supplementary Figure 3: Density plot for 81 individuals from the application cohort for the percentage of significant p-values when tested on a per sample basis for each of the 81 females against the GTEx reference cohort.

**Supplementary Tables**

Supplementary Table 1: List of samples from the validation and application cohorts with age, percentage of significantly skewed variants, predicted X-skew status, number of variants evaluated using the position, gene and global models and list of genes in patients predicted to be skewed.

Supplementary Table 2: Samples from the validation and application cohorts that were predicted to be skewed using NGS data with and without the inclusion of escape genes consisting of significantly skewed variant positions.
