# Supplementary figures and images for: Identification of skewed X chromosome inactivation using exome and transcriptome sequencing in patients with suspected rare genetic disease
Source: BMC Genomics. 2024 Apr 16;25:371. doi: 10.1186/s12864-024-10240-2 (PMC11020449; doi:10.1186/s12864-024-10240-2)

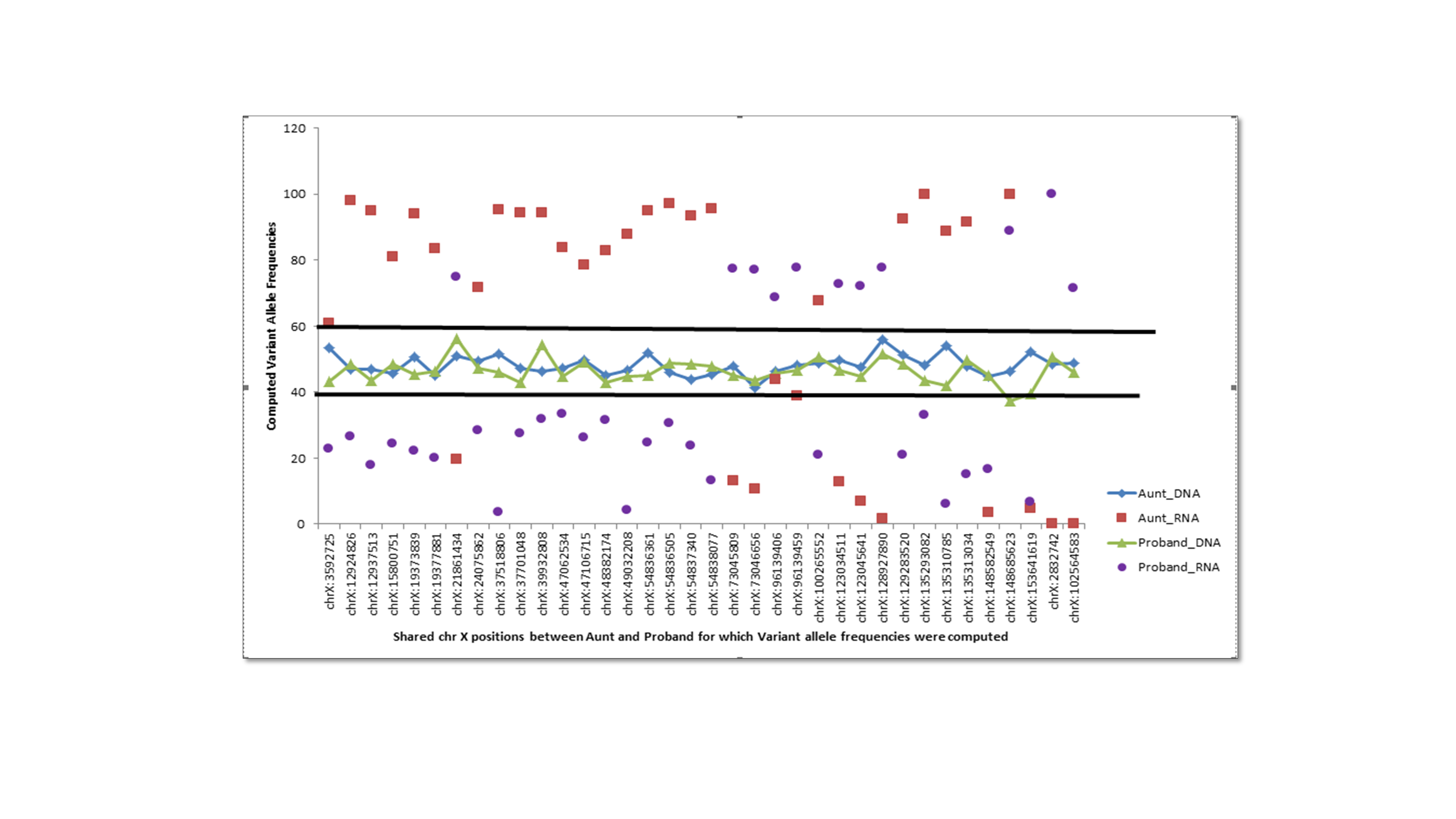

Supplement: Supplementary file 4 — Supplementary Figure 1: Computed variant allele frequency (Y-axis) for heterozygous variants shared between the proband (Sample_10) and maternal aunt (Sample_3) on the X chromosome. The variant frequencies from the maternal aunt and proband observed in the transcriptome are indicative of biased allelic expression and show a similar trend in both samples. [file 12864_2024_10240_MOESM4_ESM.png]

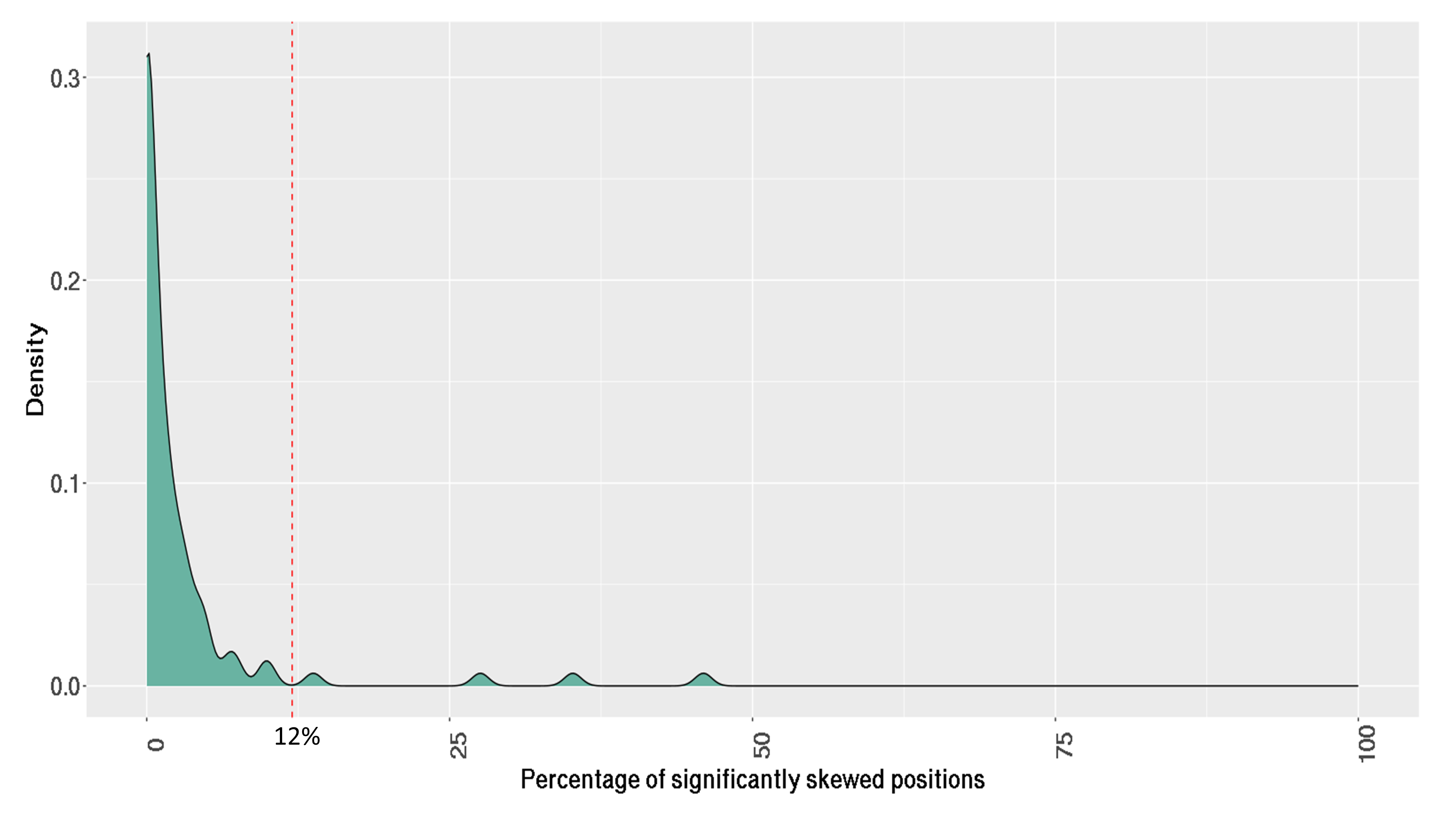

Supplement: Supplementary file 5 — Supplementary Figure 2: Density plot for 92 individuals from the validation and application cohort for the percentage of significant p-values when tested on a per sample basis for each of the 92 females against the same patient cohort. [file 12864_2024_10240_MOESM5_ESM.png]

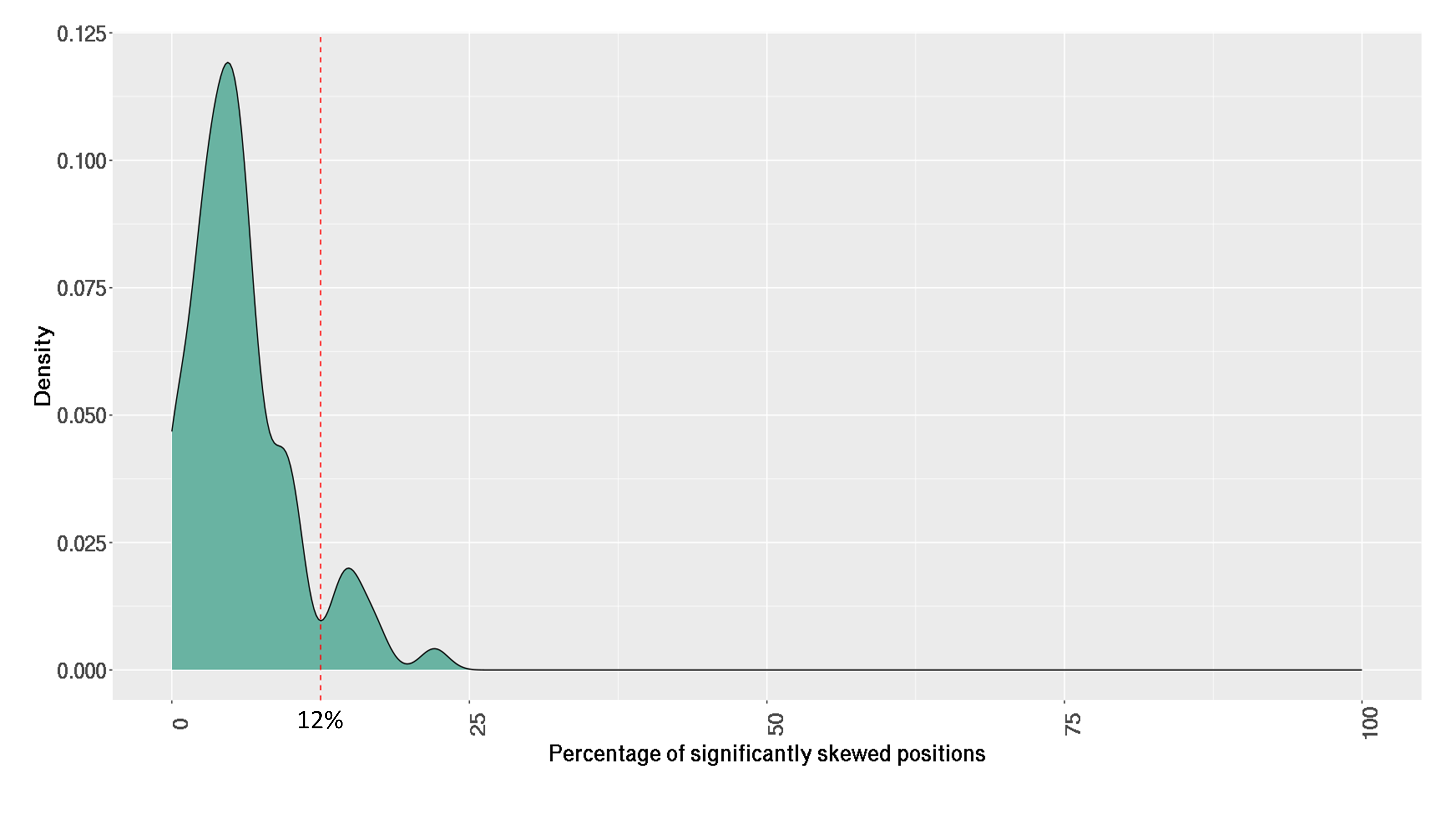

Supplement: Supplementary file 6 — Supplementary Figure 3: Density plot for 81 individuals from the application cohort for the percentage of significant p-values when tested on a per sample basis for each of the 81 females against the GTEx reference cohort. [file 12864_2024_10240_MOESM6_ESM.png]
